# Hormone-Related and Drug-Induced Osteoporosis: A Cellular and Molecular Overview

**DOI:** 10.3390/ijms24065814

**Published:** 2023-03-18

**Authors:** Li-Ting Wang, Li-Ru Chen, Kuo-Hu Chen

**Affiliations:** 1 Department of Physical Medicine and Rehabilitation, Mackay Memorial Hospital, Taipei 104, Taiwan; litingwang15@gmail.com (L.-T.W.); gracealex168@gmail.com (L.-R.C.); 2 Department of Mechanical Engineering, National Yang Ming Chiao Tung University, Hsinchu 300, Taiwan; 3 Department of Obstetrics and Gynecology, Taipei Tzu-Chi Hospital, The Buddhist Tzu-Chi Medical Foundation, Taipei 231, Taiwan; 4 School of Medicine, Tzu-Chi University, Hualien 970, Taiwan

**Keywords:** osteoporosis, hormone, estrogen, glucocorticoid, nuclear factor-κβ ligand (RANKL), osteoprotegerin (OPG)

## Abstract

Osteoporosis resulting from an imbalance of bone turnover between resorption and formation is a critical health issue worldwide. Estrogen deficiency following a nature aging process is the leading cause of hormone-related osteoporosis for postmenopausal women, while glucocorticoid-induced osteoporosis remains the most common in drug-induced osteoporosis. Other medications and medical conditions related to secondary osteoporosis include proton pump inhibitors, hypogonadism, selective serotonin receptor inhibitors, chemotherapies, and medroxyprogesterone acetate. This review is a summary of the cellular and molecular mechanisms of bone turnover, the pathophysiology of osteoporosis, and their treatment. Nuclear factor-κβ ligand (RANKL) appears to be the critical uncoupling factor that enhances osteoclastogenesis. In contrast, osteoprotegerin (OPG) is a RANKL antagonist secreted by osteoblast lineage cells. Estrogen promotes apoptosis of osteoclasts and inhibits osteoclastogenesis by stimulating the production of OPG and reducing osteoclast differentiation after suppression of IL-1 and TNF, and subsequent M-CSF, RANKL, and IL-6 release. It can also activate the Wnt signaling pathway to increase osteogenesis, and upregulate BMP signaling to promote mesenchymal stem cell differentiation from pre-osteoblasts to osteoblasts rather than adipocytes. Estrogen deficiency leads to the uncoupling of bone resorption and formation; therefore, resulting in greater bone loss. Excessive glucocorticoids increase PPAR-2 production, upregulate the expression of Dickkopf-1 (DKK1) in osteoblasts, and inhibit the Wnt signaling pathway, thus decreasing osteoblast differentiation. They promote osteoclast survival by enhancing RANKL expression and inhibiting OPG expression. Appropriate estrogen supplement and avoiding excessive glucocorticoid use are deemed the primary treatment for hormone-related and glucocorticoid-induced osteoporosis. Additionally, current pharmacological treatment includes bisphosphonates, teriparatide (PTH), and RANKL inhibitors (such as denosumab). However, many detailed cellular and molecular mechanisms underlying osteoporosis seem complicated and unexplored and warrant further investigation.

## 1. Introduction

Osteoporosis is a skeletal disorder that has a large influence on socioeconomic systems, characterized by reduced bone mass arising from the imbalance of bone resorption and bone formation that leads to changes in the microstructure of the bone [1]. The deterioration of bone microstructure results in an increased risk of fracture, giving rise to potential impairment and disability [2]. Since osteoporosis is often asymptomatic and tends to be diagnosed clinically following a fracture, which means diagnosed in the advanced stage, significant functional decline is usually inevitable after a fragility fracture [3]. Therefore, early prevention and recognition of osteoporosis are critical issues with regard to bone health [4]. Well-known risk factors of osteoporosis include old age, postmenopausal status, maternal history of osteoporosis, smoking habits, inadequate calcium intake, excessive alcohol consumption, low physical activity, and excessive glucocorticoid usage [5]. Osteoporotic fractures may cause chronic pain and limb deformity, worsen the quality of life, result in disability and further complications, and become life threatening [6]. As life expectancy expands, osteoporosis has become a major cause of morbidity and mortality in elder population, burdening the public healthcare system worldwide [7].

It is considered as primary osteoporosis in post-menopausal women and in the elderly without an obvious disease, while secondary osteoporosis is a consequence of medications or diseases [8]. Physicians possess knowledge of the association between osteoporosis and advanced age, postmenopausal status, and secondary causes, such as chronic diseases and lifestyle issues, that contribute to osteoporosis. However, they may not be aware that many widely used medications have shown relations with lower BMD and increased fracture risks, countervailing the benefits of treatment efficacy, and posing physical, economic, and psychosocial impacts on affected individuals, their families, and communities [9]. On top of that, patients often lack relevant information about the risk of osteoporosis and they might not notice it until symptomatic fragility fractures develop [10]. Thus, preventive strategies and management for osteoporosis are seldom carried out in a timely manner, consequently putting the patients in danger of osteoporotic fractures [11,12]. Therefore, being aware and alert of the potential risk of osteoporosis in different populations with specific medications is essential for clinicians to avoid treatment-related adverse events and make wise decisions during the selection of therapeutic agents [13,14].

Estrogen deficiency, following a natural aging process of the ovaries, is the leading cause of hormone-related osteoporosis for postmenopausal women [15]. On the other hand, glucocorticoid-induced osteoporosis (GIOP) remains the most common in drug-induced osteoporosis [16]. Apart from glucocorticoids, other medications contribute to an increased risk of osteoporosis as well. Common medications and medical conditions, which are related to secondary osteoporosis, include proton pump inhibitors (PPIs), hypogonadism and agents inducing hypogonadism, selective serotonin receptor inhibitors (SSRIs), chemotherapies, medroxyprogesterone acetate (MPA), antidepressants, anticonvulsants, inflammatory bowel disease, and thyroid hormone replacement or suppressive agents [9]. Additionally, as cancer treatment have advanced in recent years, the population of patients undergoing surgeries or chemotherapies that may result in secondary osteoporosis also increases [17,18].

This review aims to explore the underlying mechanisms of bone remodeling, the overall effects of estrogen deficiency on bone turnover, the pathophysiology of common osteoporosis-related medications, and medical conditions leading to secondary osteoporosis mentioned above. In addition, we make a summary of current treatment agents and manage strategies for osteoporosis. Appreciation of the cellular and molecular mechanisms in primary and secondary osteoporosis will be helpful for understanding the principles of current drug actions, and it may give us a clue to recognize new treatment targets.

## 2. Materials and Methods

### Searching Terms and Strategies in the Literature

**Figure 1:**
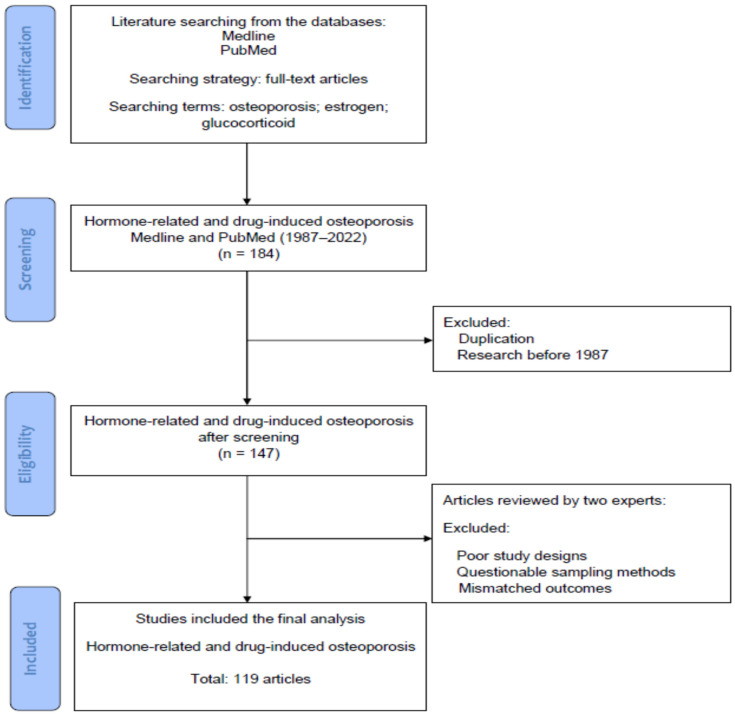
The flowchart of database searching, screening, and inclusion of the references that we selected from the literature.

The literature was searched to identify basic and clinical studies, which investigated the cellular and molecular mechanisms of osteoporosis, the pathophysiology of hormone- related osteoporosis, and drug-induced osteoporosis, along with its treatment. Figure 1 displays the flowchart of database searching, screening, and inclusion of the references that we selected from the literature. In this review, all articles were retrieved from the databases Medline and PubMed using the searching terms “osteoporosis”, “estrogen”, and “glucocorticoid” for the research topic. For screening and selection in the next stage, only full-text articles were considered for inclusion in a further analysis. In the second stage, the articles published before 1987 were excluded. Duplicated articles were also excluded. From a total of 184 articles (1987–2022) identified in the screening process, 147 potential articles met the criteria for inclusion.

Hereafter, two experts in the field independently inspected the contents of articles including demographics, research designs and outcomes, and identified eligible basic and clinical studies for inclusion. The solicited articles with poor research designs, questionable sampling methods or mismatched outcomes would be excluded at this stage. The discrepancies between the experts were discussed by their mutual communication to reach a consensus. All eligible studies were included in the review using the searching terms and strategies (identification from the database, screening of the studies, selection of potential articles and final inclusion). Finally, a total of 119 articles were collected for review from 184 articles identified in the initial search.

## 3. Cellular and Molecular Mechanisms of Bone Turnover among Osteoblasts, Osteoclasts and Osteocytes

**Figure 2:**
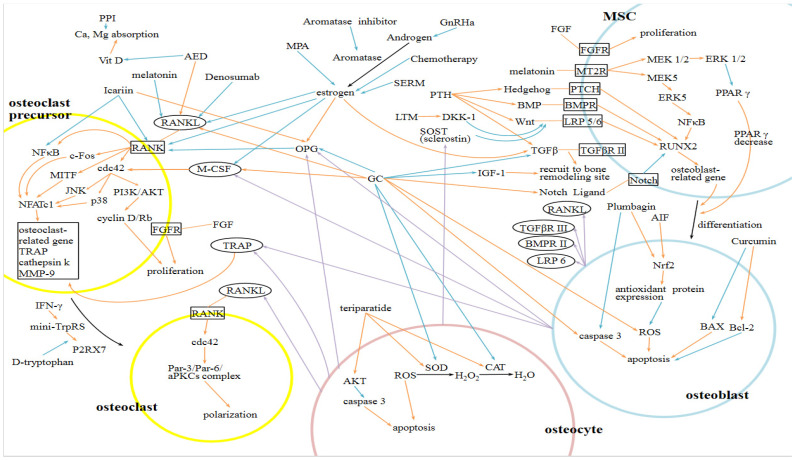
A summary of the cellular and molecular mechanisms of bone turnover (osteoblasts, osteoclasts, osteocytes, mesenchymal stem cells [MSCs], and osteoclast precursors), the pathophysiology of hormone-related osteoporosis and drug-induced osteoporosis, and their treatment. Purple line: secrete; Orange line: increase/enhance; Blue line: decrease/inhibit; *GC: Glucocorticoid; MSC: Mesenchymal Stem Cell*.

Figure 2 is a summary of the cellular and molecular mechanisms of bone turnover (osteoblasts, osteoclasts, osteocytes, mesenchymal stem cells [MSCs], and osteoclast precursors), the pathophysiology of hormone-related osteoporosis, and drug-induced osteoporosis, and their treatment.

### 3.1. Bone Remodeling

The cellular and molecular mechanisms of bone remodeling are important to the understanding of osteoporosis. The rate and extent of replacement regulate the rates of bone gain or loss, determining the three-dimensional distribution, and the amount of bone throughout the body [1]. The organization of the remodeling system profoundly influences the treatment response [1]. The bone remodeling system is regulated by multivariate factors including hormones, cytokines, paracrine factors, and gene expression. Furthermore, recent research has revealed an osteocyte-independent mechanism in bone-modeling regulation [19]. Understanding the bone remodeling system promotes the development of potential therapeutic targets.

Fracture healing is a major reason for normal bone remodeling, and a number of animal and human studies have shown the potential benefit of bone marrow-derived mesenchymal stem cells in enhancing bone repair. However, whether these cells improve fracture healing directly by differentiating into osteoblasts or indirectly by secreting paracrine factors that recruit blood vessels and the accompanying perivascular stem cells remains unclear. Among them, CD34 1 cells are enriched for endothelial/hematopoietic cells, and proved to perform a role in various bone repair models [20].

David et al. reported the treatment outcomes after 6 and 24 months of treatment with teriparatide or zoledronic acid. Given that zoledronic acid is an anti-remodeling agent by decreasing the actions of osteoclasts, teriparatide is a pro-remodeling anabolic agent that teriparatide treatment was associated with greater bone formation than zoledronic acid [21].

### 3.2. Molecular Mechanisms in Osteoclast and Osteoblast Differentiation

Osteoclast precursor cells (OCPs) are differentiated from hematopoietic stem cells. Nuclear factor of activated T-cells, cytoplasmic 1 (NFATc1) is a master transcription factor that stimulates osteoclastogenic genes, regulating diverse osteoclast-related genes, such as TRAP, cathepsin K, osteoclast-associated receptor, and matrix metalloproteinase-9 (MMP-9). The receptor activator of nuclear factor kappa-B ligand (RANKL), which is released from the surface of osteoblasts, binds to RANK on the OCPs membrane, activating the signaling pathways of nuclear factor kappa-B, c-Fos, or microphthalmia-associated transcription factor, then inducing NFATc1 and its further actions. As a key step of gene regulation, MMP-9 catalyzes histone H3 N-terminal proteolysis, leading to osteoclast-related gene expression. G9a-mediated H3K27me1 at histone H3 enables MMP-9 to localize at target genes [22].

Moreover, myeloid lineage multinucleation is a fundamental step in the osteoclast formation. Eric et al. demonstrated a truncated tryptophanyl-tRNA synthetase-dependent action of interferon-gamma (IFNγ) to promote this process in vitro, showing the potential role of the IFNγ/mini-TrpRS signaling axis in osteoporosis pathophysiology. In their study, the IFNγ induced monocyte aggregation, leading to multinuclear giant cell formation that paralleled marked upregulation of mini-TrpRS. In their study, the blockade of mini-TrpRS markedly reduced the aggregation of monocytes and the subsequent formation of multinucleation in the presence of IFNγ [23].

Cdc42, a member of small GTPases, regulates the formation and function of osteoclasts in different pathways. Cdc42 contributes to osteoclast proliferation and apoptosis rates by modulating M-CSF-stimulated cyclin D expression, triggering phosphorylation of Rb, and inducing caspase 3 and Bim. It also participates in multiple M-CSF- and RANKL-induced osteoclastogenic signals including activation and expression of the differentiation factors MITF and NFATc1. A study has reported that Cdc42-deficient osteoclasts displayed suppressed bone resorption, while osteoclasts with increased Cdc42 activity showed enhanced resorptive capacity [24].

On the other hand, mesenchymal stem cells (MSCs) differentiate into osteoblasts through signaling pathways including bone morphogenic protein (BMP), Wnt, Hedgehog, and Notch. As a primary transcription factor, Runt-related transcription factor 2 is involved in those pathways [22]. During bone resorption, the release and activation of matrix TGFβ recruits MSCs. Abnormal TGF-β signaling alters bone remodeling and causes skeletal disorders. The parathyroid hormone (PTH) modulates the signaling pathways of BMP, TGFβ and Wnts, thus directing MSC differentiation [25].

Recent studies indicate that osteocytes control the rate of bone formation and resorption by the production of RANKL and sclerostin [26]. RANKL is a TNF family member that regulates osteoclast formation, activity, and survival. Osteoprotegerin (OPG), a TNF receptor family member, prevents the effects of RANKL on bone resorption. By affecting the functions of RANKL and OPG, many hormonal and therapeutic agents have launched for the treatment of osteoporosis. Denosumab, as an anti-RANKL antibody, showed good control of bone resorption [27].

### 3.3. Hormonal and Environmental Factors

Adrenal androgen hypersecretion is related to increased bone mass. Thomas et al. have reported better proximal radial diaphyseal bone strength in children with increased prepubarchal adrenal androgen secretion, which results in reduced intracortical remodeling [28].

In contrast, glucocorticoids stimulate osteoclastogenesis via the mechanisms of increasing the expression of RANKL and CSF-1, and decreasing the expression of OPG. The inhibition of bone formation also results from decreased osteoblasts secondary to a shift in the differentiation of mesenchymal cells, and increased apoptosis or death of mature osteoblasts. Glucocorticoids further inhibit the expression of insulin-like growth factor I (IGF-1), decreasing the function of the remaining osteoblasts [29]. Additionally, a hypoxic environment is associated with inhibited osteoblast differentiation and enhanced osteoclastogenesis and bone resorptive capacity of the osteoclasts [30].

Maria et al. demonstrated melatonin-induced osteoblast differentiation and mineralization in human MSC/PBMC cocultures. Reduced osteoclastogenesis in layered cocultures as a consequence of melatonin-mediated inhibition in RANKL secretion was reported as well. Melatonin effects were mediated through MT2 melatonin receptors by coupling to both MEK1/2 and MEK. Activated MEK5 results in increases in pERK5, RUNX2, NFκB, and GLUT; activated MEK1/2 leads to increases in pERK1/2, and decreases in PPARγ, GLUT4, and IRβ. Decreased PPAR level directs MSCs differentiation to osteoblasts [31].

Current studies indicate that deficiencies in FGF signaling, a crucial signaling pathway for self-renewal of stem cells, lead to defects in bone remodeling. Expanded MSPCs and HSPCs populations after FGF-2 administration in vivo have shown its role in regulating bone formation [32].

### 3.4. Coupling between Osteoclasts (OCs) and Osteoblasts (OBs)

Extracellular matrix (ECM) proteins affect normal bone metabolism. The coupling mechanism between OCs and OBs via paracrine, autocrine, and endocrine factors allows regulation of mechanical, physical and chemical properties of bone tissue and ECM, maintaining the balance between bone formation and resorption. ECM proteins IGF-1, DCN, and ON regulate the synthesis and assembly of type I collagen, which is the main component of bone construction. TGFβ and IGF-1 act on the coupling, recruitment, maturation of OBs and OCs, and matrix formation. DCN acts on focal adhesion of osteoblast to the matrix, while OPN and BSP-2 contribute to the adhesion of osteoclast to ECM. OCN is a chemo-attractant for OBs and OCs [33].

Depending on different cell types, exosomes transmit signals regulating the differentiation, recruitment, and activity of cells involved in bone remodeling. MSC-derived exosomes favor osteogenesis. Osteoblast-derived exosomes implicate four osteogenesis-related pathways: Rho GTPase binding, integrin, and mTOR and EIF2 signaling, via the actions of some key exosomal proteins including ephrin-B1, transforming growth factor beta receptor 3, low-density lipoprotein receptor-related protein 6, bone morphogenetic protein receptor type 1, and SMURF1. Osteocytes produce vesicles containing osteoclastogenesis-regulating factors, such as TRAP, RANKL, and osteoprotegerin (OPG) [34].

### 3.5. Gene Expression

Hypomethylation of the promoter regions of specific osteogenic genes, such as RUNX2 and OCN in BMSCs leads them into osteoblasts lineage, while hypomethylation of the adipose tissue-related gene PPAR-g2 in ASCs directs them into adipocytes. Recent studies indicate that bone remodeling processes are epigenetically regulated by DNA methylation and histone post-translational modifications. DNA methylation status modulates genes associated with osteoblast and osteoclast differentiation, including RANK/RANKL/OPG, RUNX2, OSX, OCN, ALP, and Wnt pathways [35]. In a study, Icariin was reported to promote osteoblast activity and inhibit osteoclast activity in vitro by enhancing the expression of OPG and RANKL gene, and reducing the expression of NFkb [36].

### 3.6. Other factors which Influence the Bone Turnover and the Balance of Osteoblassts and Osteoclasts

#### 3.6.1. Secretome Related to Senescence and Bone Loss

Senescence-associated secretory phenotype (SASP) is a unique secretome found in senescent cells, which mainly contributes to recruitment of immune cells for the clearance of senescent cells, and it was recently found to perform a role in osteoporosis. A DNA damage response (DDR) is triggered by telomere dysfunction and an increase in ROS trigger, leading to activation of NF-κB pathway and elevated SASP secretion. The production of skeletal-aging-related SASP is most identified in bone marrow myeloid cells and osteocytes, including matrix modifying enzymes, and chemokines and interleukins working in a manner of autocrine, paracrine, or endocrine. These SASP factors facilitate suppression of osteoblast function and activate osteoclastogenesis [37].

#### 3.6.2. Secretome of Osteoblasts Promotes Cell Growth in Osteoblasts

The secretome of osteoblasts includes progranulin and it acts as an autocrine growth factor. Progranulin promotes cell growth in osteoblasts via activation of the MAPK (ERK1/2) signaling pathway and enhances the survival of osteoblasts. Risedronate has been found to increase the expression and secretion of progranulin to prevent osteoporosis [38].

#### 3.6.3. Secretome of MSCs in Ovariectomized Rats Promotes Bone Resorption

Mesenchymal stem cells (MSCs) secrete factors regulating bone homeostasis, and secretome changes lead to tilting of bone homeostasis toward pro-resorptive status. MSCs of ovariectomized rats could release secretome containing Activin A, CXCL1, CX3CL1, MCP-1, TIMP-1, and TNF-α, that facilitate bone resorption via inhibiting proliferation and differentiation of osteoprogenitor cells and promoting fusion of osteoclasts. The partial dependence of N-cadherin in secretion of the aforementioned factors was also observed [39].

#### 3.6.4. Extracellular Vesicles in Bone Homeostasis

Extracellular Vesicles (EVs) are small nanoparticles with a lipid bilayer released by exocytosis, containing various intracellular components, such as RNAs, lipids, and proteins, and they enter the target cells by endocytosis. In brief, EVs are demonstrated to be part of the cellular signaling network and perform a role in the crosstalk between bone cells and immune cells, becoming a potential treatment approach in bone diseases. Monocyte-Evs enhance osteogenic differentiation via facilitating RUNX2 and BMP-2 secretion in MSCs. Osteoclast-Evs regulate osteoblasts formation. MSC-Evs promote the recruitment and activation of macrophages [40].

#### 3.6.5. The Alteration of Osteocytes Secretory Profile (Secretome and Extracellular Vesicles in Osteocytes) in Response to Mechanical Stress

Osteocytes respond to mechanical loading and enhance bone formation by releasing various factors, including NO, PGE2, RANKL, OPG, and M-CSF in the form of extracellular vesicles (EVs). The release of osteocyte-produced sclerostin is decreased under mechanical loading, blocking its inhibitory effect on Wnt-mediated bone formation, while increased release of sclerostin in unloaded bone leads to bone loss. The alteration in the secretome of osteocytes in response to fluid shear stress has been demonstrated, indicating several factors involved in mechano-transduction of bone. Osteocytes-derived mechanically activated EVs perform a role in MSCs osteogenesis and recruitment, showing a mechanism in machano-adaptation of bone and emerging as a potential delivery of therapeutic agents [41].

## 4. Cellular and Molecular Mechanisms of Hormone-Related and Drug-Induced Osteoporosis

Hormone-related and drug-induced osteoporosis has become a significant health problem that physicians should be aware of. Aside from glucocorticoids, common medications, including proton pump inhibitors (PPIs), selective serotonin receptor inhibitors (SSRIs), and anticonvulsants, are also related to osteoporosis. Thiazolidinediones, medroxyprogesterone acetate (MPA), aomatase inhibitors (AIs), androgen deprivation therapy (APT), heparin, calcineurin inhibitors, and some chemotherapies are reported to have deleterious effects on bone health [9]. The underlying mechanisms, pathways, and related mediators of common medications which induce osteoporosis are shown in Table 1.

### 4.1. Hormone Related Osteoporosis (HROP)

Estrogen promotes the apoptosis of osteoclasts and inhibits osteoclastogenesis via several pathways. Estrogen not only stimulates the production of OPG, but also reduces the differentiation of osteoclasts by suppressing IL-1 and TNF, therefore inhibiting the release of M-CSF, RANKL, and IL-6. Estrogen promotes the apoptosis of osteoclasts via the effect of TGF-β. Estrogen deficiency leads to the uncoupling of bone resorption and formation, which means an increased osteoclastic resorption without a corresponding osteoblastic activity. The osteoblastic activity fails to catch up with increased osteoclastic resorption, therefore resulting in greater bone loss. RANK ligand (RANKL) appears to be the critical uncoupling factor that enhances osteoclastogenesis. During estrogen deficiency, both the production of TNF and the sensitivity to IL-1 of stromal cells increase, stimulating stromal cells to release IL-6, M-CSF, IL-11, GM-CSF, and TGF. The cascade leads to the secretion of RANKL from osteoblasts, binding to RANK on osteoclasts, and promoting osteoclast development. On the other hand, osteoprotegerin (OPG) is an antagonist against RANKL secreted by osteoblast lineage cells, and it contributes to the anti-resorptive actions of estrogen [15].

In postmenopausal women, the markers of bone turnover were found higher in females with osteoporosis than those in the control group. Noted in another study, bone turnover markers had an inverse relationship with spine BMD, while spine BMD correlated positively with serum estradiol levels [42]. In contrast, lower serum lncRNA SNHG1 level due to down-regulation after menopause was found in postmenopausal osteoporotic women. It may be a potential biomarker for diagnosis or treatment target for postmenopausal osteoporosis [43]. While the absolute fracture risk determined the intervention threshold, Abrahamsen et al. discovered that the fracture risk in postmenopausal women was higher than the reported risk predicted by the risk assessment tool [44]. Boschitsch et al. indicated that the proportion of osteoporotic population increased with advancing age. However, the risks of osteoporosis and related fractures in middle-aged women were under-recognized and BMD measurement might not be sufficient to identify patients at risk of fragility fractures [45]. The length of time after menopause and BMI were established as the most significant factors associated with osteoporosis. Nonetheless, these results indicated that rather than age, estrogen deficiency performed the crucial role in osteoporosis [46].

In postmenopausal women with rheumatoid arthritis, older age, low BMI, and high cumulative dosage of glucocorticoids were associated with an elevated risk of osteoporosis [47]. Akdeniz found body weight and the age of menopause were strongly associated with osteoporosis [5]. The correlation between low whole body fat and low BMD was found in postmenopausal women with osteoporosis [48]. Compared to healthy postmenopausal patients, patients with osteoporosis were not different in age and age at menarche. Years of menstruation significantly differed in two groups as a protective factor against osteoporosis [3]. Another study also confirmed that the longer length of menstruation was related to lower incidence of osteoporosis, suggesting that cumulative estrogen exposure determined the protective effect against osteoporosis [49]. Several studies had concluded the association between the length of menopause time and osteoporosis, indicating the influence of cumulative estrogen exposure [50,51].

Early menopause is associated with increased fragility fracture risk and mortality [4]. Some single nucleotide polymorphisms (SNPs) within osteoporosis candidate genes, such as ESR1 and MHC, seem to have an influence on the age of natural menopause [52]. When it comes to the factors affecting menopause, surgical menopause is associated with a higher osteoporosis rate than natural menopause [18]. Comparing to those in women with natural menopause, increased cardiovascular risk factors are found in women with surgical menopause [53].

To sum up, risk factors of postmenopausal osteoporosis include advanced age, a family history of osteoporosis, lifestyle variants (poor nutrition, insufficient physical activity, smoking, and heavy alcohol consumption), and low BMI. Subsequent hip and spine fractures contribute to the high mortality in postmenopausal women with osteoporosis. Common risk factors of fragility fractures are advanced age, low BMD, and previous fractures. Interestingly, longer sleep duration and longer napping time during daytime seem to be associated with osteoporosis in postmenopausal women [54]. Nonpharmacologic management includes balanced diet, calcium and vitamin D intake or supplement, smoking cessation, avoiding alcohol consumption, adequate exercise, and fall prevention. Pharmacologic therapy includes bisphosphonates, parathyroid hormone (PTH), estrogen, and calcitonin [55]. Olive oil supplement has shown anti-osteoporosis and antioxidative effects in vivo; however, further studies are required to determine the mechanisms [56].

A survey which inspected the knowledge and behavior of bone health issues conducted in women with early menopause reported poor awareness of postmenopausal osteoporosis, and inadequate calcium intake and exercise. Education could eliminate the knowledge gap [10]. More than half of perimenopausal women struggled with obstetric or gynecologic issues including postmenopausal osteoporosis. Thus, it is critical for primary care providers and patients to understand the issues regarding the risk of osteoporosis in menopause and its corresponding treatment [57].

As mentioned above, treatment options with valid evidence include calcium and vitamin D supplement, bisphosphonates, denosumab, and PTH. Calcitonin, estrogen, and estrogen plus progestin therapy inhibit bone resorption. Teriparatide (PTH) increases bone formation [58].

Hormone replacement therapy is generally considered a second-line treatment option, while it performs a role in the management of perimenopausal women with menopausal symptoms and elevated fracture risks [59]. A low protein diet aggravates postmenopausal bone loss, and its negative effect on bone health can be eliminated by HRT [60].

Estrogen therapy requires progestin to reduce the risk of endometrial hyperplasia; otherwise, the risks of irregular bleeding and breast cancer increase consequently. Although SERM itself may induce osteoporosis, the combination of conjugated equine estrogen and bazedoxifene, a third-generation SERM, has been developed to prevent postmenopausal osteoporosis and vasomotor symptoms. As SERMs can act as an antagonist on endometrial tissue, the estrogen plus bazedoxifene strategy avoids progestin use and related adverse effects [61,62].

In patients with gynecological cancers, cancer treatment may result in loss of ovarian function and eventual early menopause [63]. The patients who experience iatrogenic menopause secondary to various cancer treatments have increased recently as cancer treatments advance. While these women are fragile due to the primary disease and consequent treatment toxicity, early menopause may further threaten their physical and psychological health. Thus, physicians should pay attention to the prevention and treatment of potentially severe and premature osteoporosis in this population. Since hormone therapy has a risk of stimulating tumor growth, non-hormonal therapies are considered preferential in these patients [63]. Several bisphosphonates including clodronate, risedronate, and zoledronic acid have shown positive effects on the BMD of patients with menopause related to chemotherapy and LHRH agonists [64].

Life expectancy continues to increase, therefore, the postmenopausal population expands with longer deprivation of estradiol and progesterone, resulting in increased postmenopausal osteoporosis and other related disorders. A new method has been proposed to deal with postmenopausal disorders via ovarian cryopreservation in youth and grafting of ovarian tissue after menopause. While lacking evidence, it is technically available now and may be a potential solution in the future [65].

### 4.2. Drug Induced Osteoporosis

#### 4.2.1. Glucocorticoids Induced Osteoporosis (GIOP)

Mariateresa et al. reported a decreased bone density in young patients with congenital adrenal hyperplasia receiving glucocorticoids compared to healthy controls [66]. Glucocorticoid induced osteoporosis is a multifactorial mechanism, including decreased bone formation and increased bone resorption.

Glucocorticoids decrease the differentiation of osteoblasts by increasing the production of PPAR-2, a transcription factor promoting stromal cells to differentiate into adipocytes rather than osteoblasts. The Wnt signaling pathway performs a role in the regulation of osteoblast proliferation and differentiation. Upregulation of the expression of Dickkopf-1 (DKK1) in osteoblasts was reported in dexamethasone therapy, inhibiting the Wnt signaling pathway by interacting with the Wnt co-receptors LRP5 and LRP6. This effect is found to be time-dependent and dose-dependent. Glucocorticoids directly suppress bone protein matrix synthesis via modulating the expression of Runx2/Cbfa1. Cortisol is also reported to decrease Insulin-like growth factor-I (IGF-1), which is a stimulator of bone formation. Moreover, suppressed osteoblast activity is observed during glucocorticoid treatment.

Excessive glucocorticoids not only reduce the production of osteoblasts, but also shorten the lifespan of osteoblasts in a dose-dependent manner. Caspase 3 is a key mediator of apoptosis, and it is involved in multiple apoptotic signaling pathways. Activation of caspase 3 contributes to glucocorticoid-induced bone cell apoptosis [16]. It is reported that glucocorticoids induce the differentiation of osteoblast via multiple pathways, such as parathyroid hormone—stimulated cAMP synthesis, osteopontin, bone sialoprotein, alkaline phosphatase, and mineralized bone nodule formation. However, excessive glucocorticoids have also been demonstrated to reduce type I collagen synthesis, osteocalcin production, mRNA levels, the expression of IGF-1, and selected IGF-binding proteins which enhance IGF activity [67]. In vitro, osteoblast viability is increased under physiological doses of glucocorticoid, while higher doses of glucocorticoid lowers osteoblast viability by inducing apoptosis. Glucocorticoids are also related to reduced expression of hypoxia-inducible factor and vascular endothelial growth factor (VEGF), with consequent loss of osteoblasts and bone mass. Glucocorticoids stimulate the Notch signaling pathway, which is associated with decreased bone production [68].

Glucocorticoids promote osteoclast survival by enhancing the expression of receptor RANKL, which is important in osteoclastogenesis, and inhibiting the expression of its soluble decoy receptor, osteoprotegerin (OPG). Enhanced expression of colony stimulating factor-1, which results from excessive glucocorticoids, further induces osteoclastogenesis in the presence of RANKL. The aforementioned mechanisms lead to accelerated bone resorption [16]. In past studies, the macrophage-colony stimulating factor and RANKL were proved essential in triggering and sustaining osteoclastogenesis. Furthermore, Faienza et al. identified a T cell-dependent spontaneous osteoclastogenesis, without adding macrophage-colony stimulating factor and RANKL, in patients with 21-hydroxylase deficiency under chronic glucocorticoid therapy. Compared to those in the control group, T cells from these patients expressed higher levels of RANKL and lower levels of osteoprotegerin (OPG) [69].

In aspects of hormones, glucocorticoids pose an impact on calcium excretion and absorption, promoting hypocalcemia, with secondary increased parathyroid hormone (PTH), ending in increased bone resorption. Glucocorticoids also contribute to bone loss by inhibiting the release of gonadotropin [70].

Glucocorticoids induce osteoporosis in a dose-dependent and time-dependent pattern. Both current and cumulative doses are associated with increased fracture risks. A dosage of 10-15 mg of prednisone per day approximates the threshold of significant elevated fracture risks [71].

A survey conducted in Massachusetts has revealed that a considerable proportion of patients under long-term glucocorticoid therapy do not receive preventive treatment against osteoporosis [72]. Among patients with osteoporosis, those treated with glucocorticoids seem to develop minimal trauma fractures at higher bone mineral density (BMD) than those with other primary or secondary causes of osteoporosis. Thus, using BMD as a parameter to predict fragile fractures may underestimate the fracture risk in glucocorticoid-induced osteoporosis, resulting in under-recognition and under-treatment of the disease prior to fractures [73]. Since using BMD as a sole parameter underestimates the fracture risk in glucocorticoid-induced osteoporosis, Robert et al. have suggested that assessment should include the FRAX calculation and images of the spine [71].

Leonardo et al. have demonstrated a novel measurement of cortical bone change using reference point indentation technique. This method is sensitive to detect the treatment response of glucocorticoid-induced osteoporosis [74].

Other risk factors related to fractures have been identified, including age, BMI, and female sex. Several longitudinal studies have demonstrated reversible bone loss in glucocorticoid-induced osteoporosis, where bone density increases following the discontinuation of glucocorticoids [75].

#### 4.2.2. Aromatase Inhibitor Associated Osteoporosis

As an important part of standard care for postmenopausal women with hormone receptor positive breast cancer, aromatase inhibitors (AIs) cause decreased BMD, and increased fracture risks. In healthy postmenopausal women, exercise programs with vitamin D and calcium supplement prevent osteoporosis by reducing bone resorption. This strategy is investigated to against the osteoporotic effect in postmenopausal women prescribed AIs for the treatment of breast cancer [76].

Comparing to healthy controls, bone loss occurs at a 2-fold higher rate with increased fracture risks in postmenopausal women receiving aromatase inhibitor therapy, indicating a different pattern from postmenopausal osteoporosis. Thus, identifying and managing osteoporosis and fracture risks are essential in patients with breast cancers who are under AI therapy. Nitrogen-containing bisphosphonates have been studied as a treatment option for aromatase inhibitor-associated bone loss [77].

#### 4.2.3. Gonadotropin Releasing Hormone Agonist (GnRHa) Associated Osteoporosis

Androgen deprivation therapy (ADT) with gonadotropin releasing hormone agonist (GnRHa) is important in patients with prostate cancers. Yuasa et al. demonstrated the similar prevalence of osteoporosis in prostate cancer patients treated with ADT and hormone-naive patients in the Japanese population, while other studies found increased osteoporosis in patients under ADT compared to control groups. The population differences may be attributed to genetic and hormonal factors [78]. Miyaji et al. investigated the bone resorption and compensatory bone formation in patients under GnRHa therapy via examining the changes of biochemical markers. As a marker of bone resorption, carboxyl-terminal telopeptide of type I collagen (ICTP) rose significantly after 6 months of the prescription of GnRHa. Carboxy-terminal pro-peptide of human type I procollagen (PICP), the marker of bone formation, increased significantly 1 year after the start of treatment, after the increase in ICTP. A significantly lower BMD was also observed after 2 years of GnRHa treatment [79]. In an analysis of men surviving at least five years after the diagnosis of prostate cancer, increased fracture risks were reported in patients who had received ADT for 12 months in a dose-dependent manner as compared with those not receiving ADT [80].

Long-term ADT is a well-established treatment for localized high risk prostate cancers. Though evidence remains controversial, long-term ADT has been thought to be associated with bone loss and fracture risks due to consequent hypogonadism. Supplementation of calcium with vitamin D is proved to be sufficient in preventing bone mineral density loss in patients with prostate cancers who receive long term ADT [81].

Osteoporosis in men is also a noteworthy issue that may be underestimated. Several pharmacologic interventions, such as bisphosphonates, estrogen receptor-binding drugs, calcitonin, fluoride, and statin agents, have been investigated to reduce the risk of osteoporosis. Other managements, including weight-bearing exercise and smoking cessation, may also be beneficial. Potentially effective approaches to manage or prevent osteoporosis exist and patients should be informed. After discussion with patients, the intervention should be started before ADT prescription to achieve maximal advantage [82].

Combined therapy using a single-agent tamoxifen or aromatase inhibitor with ovarian function suppressing agent (OFS), such as GnRHa, has emerged as an adjuvant therapy for premenopausal women with breast cancers. The potential effect of OFS has been examined. Compared to patients without OFS treatment, those with adding OFS to another agent are at a higher risk for osteoporosis [83].

As a treatment for premenopausal women with endometriosis, GnRHa inhibits pituitary gonadotropin release via down-regulation of receptors, and results in inhibition of ovarian estrogen production. Thus, hypoestrogenic state leads to suppression of endometriotic implants and a decrease in pain. However, reduced estrogen levels cause major side effects, such as osteoporosis. An analysis using transiliac biopsies has revealed that GnRH analog treatment for endometriosis is related to increased bone turnover resulting from estrogen suppression. A study revealed that estrogen suppression enhanced the activity of Tartrate-resistant acid phosphatase (TRAP) and osteoclasts. Compensatory increased osteoblast activity was also observed [84].

The side effects resulting from low estrogen concentration are often reversible when short-term GnRHa therapy is discontinued, and ovarian function recovers. Reduced BMD after 6-month or shorter GnRHa is found reversible, but irreversible and advanced bone loss occurs in prolonged therapy of up to 12 months, causing further adverse events and poor compliance. To manage the secondary hypoestrogenic side effects of GnRHa, add-back therapy is developed, combining selected pharmacologic agents with GnRHa to maintain therapeutic efficacy and minimize the hypoestrogenic effects. Norethindrone acetate 5 mg per day, with or without estrogen, appears to be effective to allow prolonged GnRHa therapy and reduce hypoestrogenic side effects. Add-back therapy should be initiated in patients requiring extended GnRHa usage beyond 6 months, and it may be an option in patients with short-term GnRHa usage [85].

#### 4.2.4. Chemotherapy Induced Osteoporosis

Chemotherapy-related osteoporosis is attributed to direct and profound damage to ovarian tissues resulting from chemotherapeutic agents. Consequently, secondary ovarian failure occurs, leading to decreased intestinal calcium absorption, increased renal calcium excretion, increased PTH secretion, increasing bone turnover, and ending in osteoporosis. Several chemotherapeutic agents are associated with damage to ovaries, especially alkylating cytotoxics. Methotrexate and doxorubicin are also reported to affect BMD in animal models. Poor intake of calcium and vitamin D due to chemotherapy may further contribute to bone loss. Administration of estrogen, bisphosphonates, calcium and vitamin D supplements are effective in preventing chemotherapy-related osteoporosis [17].

#### 4.2.5. Selective Estrogen Receptor Modulators (SERMs) Related Osteoporosis

Estrogen receptors (ERs) are divided into two categories: type α and type β. The distribution and ratio of ERα and ERβ varies in different tissues. The tissue response to estrogen differs depending on the type of ER receptors activated. When estrogen binds to ERs, dimerization of ERs starts before it binds to the estrogen responsive DNA sequence. The product of dimerization can be an ERα-ERα homodimer, an ERα-ERβ heterodimer, or an ERβ-ERβ homodimer. The variety of ER dimers may contribute to different tissue-specific responses to estrogens. Transcriptional activator proteins are then recruited, modulating tissue reactions in response to estrogen. In brief, complicated tissue response to estrogen is based on different ER subtypes, ER dimerization and tissue-specific transcriptional proteins. Similarly, the effects of SERMs may be difficult to predict due to the complexity of tissue-specific response to estrogens.

For instance, tamoxifen behaves as an estrogen antagonist at hypothalamic level and increases FSH, while in endometrium, it acts as an estrogen agonist. Tamoxifen has an estrogen agonist activity in bone tissue, lowering bone resorption, and maintaining BMD.

The effect of bazodoxifene on ER degradation has been demonstrated in animal studies, leading to down regulation of ER degradation. This unique reaction makes bazodoxifene a good antiresorptive agent on bone. A new therapy termed “tissue selective estrogen complex” combining bazodoxifene and conjugated equine estrogens has been developed, showing a significantly increased BMD [86].

#### 4.2.6. Proton-Pump Inhibitor (PPI) or Diabetes Mellitus (DM) Related Osteoporosis

Peptic ulcer disease (PUD) is a common comorbidity of diabetes mellitus, causing long-term proton-pump inhibitor (PPI) usage in a large proportion of patients with diabetes mellitus. Observational studies showed increased risk of osteoporosis and hip fracture related to PPI therapy in DM patients [87]. Since PUD-related treatment was viewed as a risk factor for osteoporosis, Wu et al. conducted a survey among Asian population to investigate the risk of osteoporosis related to PPI use in PUD patients. A higher rate of osteoporosis was found in patients using PPI compared to those who did not [88]. A significantly elevated risk of osteoporotic fracture was also reported in patients under PPI treatment for more than 7 years in a Canadian study [89].

Further research demonstrated an increased prevalence of osteoporosis in patients under PPI therapy. However, the pathogenesis remained unclear. Several hypotheses were proposed, but failed to be confirmed, thus specific interventions to manage osteoporosis in patients with PPI are still lacking [90]. Although multiple studies have demonstrated the linkage between PPI and osteoporosis, there is a lack of well-established pathological mechanisms. Recently, Targownik et al. investigated the causation between long-term PPI use and change in BMD or micro-structure predisposing to fractures. Paradoxically, areal BMD, volumetric BMD, and biomarkers of bone metabolism failed to show any significant difference between patients with long-term PPI treatment and control groups [91].

#### 4.2.7. Anti Convulsion Drug Induced Osteoporosis

Parveen et al. demonstrated the osteoporotic effect of anti-epilepsy drug (AED) in normal and ovariectomised rats. AEDs were related to reduced BMD at lumbar vertebrae and femur epiphysis, and the reduction was found to be more significant in ovariectomised rats compared to normal rats. Elevated serum RANKL, DKK-1, and sclerostin levels, and decreased vitamin D, and P1NP level were observed after AEDs treatment, which intimated the possible mechanism of osteoporotic effect of AED, via the Wnt signaling pathway. Synergically, estrogen deficiency reducing bone formation via decreased P1NP, and increased sclerostin and enhancing bone resorption via increased RANKL may contribute to further BMD reduction in ovariectomised rats [92].

Ensrud et al. reported an accelerated bone loss in elderly women treated with AEDs. The rate of bone loss was found 1.7-fold faster in continuous AED users than in partial users [93]. Ahmad et al. estimated the fracture risk in epilepsy patients and reported an increased fracture risk in patients using AED compared to nonusers. Among AED users, the fracture risk increased with prolonged AED therapy; female users were at a higher risk of falls when compared to female nonusers [94]. Another cohort study suggested that AED was also associated with reduced BMD at hip in elderly men [95]. However, it was estimated that less than 30% of these patients were aware of the potential risk of bone loss, fractures, and falls related to AEDs [94].

#### 4.2.8. Medroxyprogesterone Acetate (MPA) Associated Osteoporosis

Decreased bone density was found to be associated strongly with DMPA (Depot medroxyprogesterone acetate) exposure in women, and the recovery of bone density was observed 30 months after discontinuation [96]. Cromer reported that young women might fail to achieve optimal peak bone mass due to use of DMPA as a contraceptive [97].

DMPA treatment in adolescents and young women was found to be related to bone loss after 1 to 2 years of usage, with slow and partial recovery after discontinuation. This finding raised a concern that these adolescents and young women might be at risk of irreversible decrease in bone density. Thus, the United States Food and Drug Administration issued a black box warning on the long-term use of DMPA in 2004 and suggested that all women using DMPA should maintain adequate calcium intake and avoid other risk factors of osteoporosis, such as smoking or caffeine [98].

## 5. Medication for Treating Osteoporosis

Table 2 presents a timeline of medications available for the treatment of osteoporosis. There are several drugs that target osteoclasts and their activity. For example, bisphosphonates and denosumab are two classes of drugs that are commonly used to treat osteoporosis. Bisphosphonates work by binding to bone and inhibiting osteoclast activity, while denosumab is a monoclonal antibody that targets a protein called RANKL, which is required for osteoclast differentiation and activity.

### 5.1. Bisphosphonates

Common bisphosphonates include alendronate, risedronate, minodronate, and intravenous zoledronic acid. In comparison with calcitriol and vitamin D, alendronate showed better efficacy in preventing and managing GIOP [99]. In patients with GIOP, alendronate treatment significantly increased BMD in the femoral neck and lumbar spine. However, it seemed that alendronate failed to reduce the risk of osteoporotic fractures [100]. Another study also suggested that alendronate was not associated with reduced fracture risks, though it increased BMD in the lumbar, hip, and trochanter significantly [101]. As mentioned above, minodronate, a third-generation bisphosphonate, was also related to increased lumbar BMD and reduced bone resorption markers in patients with GIOP [102].

As bisphosphonates have been proved as an effective treatment of osteoporosis, the compliance of bisphosphonates was investigated. Patients using bisphosphonates weekly were more adherent than those with daily doses. The gastrointestinal adverse events were associated with discontinuation of the medication [103]. Reid et al. assessed the efficacy of intravenous zoledronic acid and oral risedronate in patients with GIOP. A single dose of intravenous zoledronic acid infusion was similar to daily dose of oral risedronate in increasing lumbar spine BMD, showing a non-inferior prevention and treatment effect of GIOP. For the patients’ convenience, a single dose of intravenous zoledronic acid infusion might be more acceptable than daily risedronate to patients with glucocorticoid use [104].

In addition to glucocorticoids, ADT is another major cause of secondary osteoporosis. Campbell et al. demonstrated the effectiveness of zoledronic acid for improving BMD in prostate cancer patients under ADT [105].

### 5.2. Parathyroid Hormone (PTH)

Although bisphosphonates are effective in treating osteoporosis by decreasing bone resorption, parathyroid hormones stimulate bone formation prevents osteoporosis in a way different from bisphosphonates. Compared to HRT alone, HRT plus injections of aminoterminal fragment of human PTH 1–34 [hPTH(1–34)], which is the bioactive portion of human PTH, showed a markedly increased BMD in lumbar spine and hip in postmenopausal women with GIOP. Moreover, the therapeutic effect persisted 6 months after discontinuing the treatment [106].

Teriparatide is a parathyroid hormone formed by recombinant DNA, consisting of the first (N-terminus) 34 amino acids of parathyroid hormone. In vitro, decrease in ROS and caspase-3 were found in cells in the dexamethasone plus teriparatide group compared with cells that were only exposed to dexamethasone, indicating that teriparatide prevented the apoptosis effect induced by dexamethasone via promoting AKT pathways [107]. In vivo, teriparatide showed a positive relationship with increased BMD compared to the placebo. Moreover, lower fracture rates were found in the teriparatide group than the alendronate group. Unlike antiresorptive agents, the treatment effect of teriparatide did not require previous bone resorption [108]. For men with GIOP, both teriparatide and risedronate increased BMD, while teriparatide showed more improvements in spinal BMD than risedronate [109]. As the reduction in osteoporotic fractures associated with teriparatide and denosumab usage was demonstrated, Hsu et al. suggested that for patients at risk of osteoporosis, if bisphosphonates were contraindicated, teriparatide and denosumab should be considered as an alternative [110].

According to the level of evidence, American College of Rheumatology (ACR) guidelines have assigned an A rating to alendronate and risedronate, and a B rating to zoledronate and teriparatide for the treatment of GIOP. GIOP leads to marked bone loss within the first 6–12 months of glucocorticoids therapy, and the risk of fracture increases rapidly within the first 3 months. Thus, early initiation of bisphosphonate or teriparatide therapy for those whose glucocorticoids treatment duration is expected to exceed 3 months should be emphasized.

While clinical trials for management of GIOP last no more than 3 years, a considerable portion of patients require long-term glucocorticoids therapy. Thus, further evaluation and possibly additional treatment for those requiring longer-term therapy remain unsolved. Considering young individuals, the ACR guidelines recommend starting treatment for those with fragile fractures. Nevertheless, osteoporosis treatment may also be necessary for premenopausal women who suffer from a significant decline in BMD after long-term glucocorticoid therapy. For patients who are pregnant or planning a pregnancy, bisphosphonates are concerned about the risk of crossing the placenta and posing an impact on fetal health. On the other hand, teriparatide is totally eliminated when therapy is terminated, and therefore, seems not to affect fetal bone growth. Denosumab has a relatively short half-life compared to bisphosphonates, and it may be another treatment option for premenopausal women with GIOP who plan a future pregnancy [111].

### 5.3. Potential Treatment Target in the Future

Alpinumiso flavone A (AIF), a naturally occurring flavonoid compound, was found to inhibit dexamethasone-induced apoptosis. AIF promoted the Nrf2 signaling pathway, suppressing dexamethasone induced ROS production, and thus had a protective effect against GIOP [112].

In vivo, curcumin administration for 60 days reversed the apoptosis of osteoblast induced by dexamethasone via reducing the ratio of Bax/Bcl-2, caspase-3 level, and promoting the expression of p-ERK1/2 [113].

Sclerostin, expressed by the Sost gene, is recognized as an inhibitor of the Wnt/β-catenin signaling pathway that performs a vital role in promotion of bone formation and suppression of bone resorption. Sato et al. investigated the protective effect against glucocorticoid-induced bone loss of the Wnt/β-catenin pathway using Sost gene-deleted mice. Under basal conditions, higher bone mass, increased bone formation and unchanged resorption were found in Sost-deleted mice. However, under excessive glucocorticoids, Sost/sclerostin deficiency did not prevent glucocorticoid-induced reduction in bone formation. The researchers also discovered that activation of the Wnt/β-catenin pathway due to Sost/sclerostin deficiency preserved bone mass by opposing bone resorption effects caused by glucocorticoids [114].

Zhang et al. demonstrated that the protective effect against GIOP of plumbagin was associated with reducing ROS and apoptosis markers. Plumbagin activated nuclear factor erythroid-derived 2-like 2 (Nrf-2) pathways, a transcription factor regulating the expression of antioxidant proteins, reducing the oxidative stress induced by glucocorticoids. Inhibition of NF-kB expression also contributed to the protective effect of plumbagin, reversing apoptosis resulting from glucocorticoids [115].

The gene encoding glucocorticoid-induced leucine zipper (GILZ) is identified as a greatly influenced gene in Cushing’s syndrome. The role of GILZ in GIOP was studied in patients with Cushing’s syndrome and the outcome revealed that GILZ may contribute to GIOP via manipulating osteoblast maturation and bone turnover [116].

Compared with cell-based therapy, the therapy employing extracellular vesicles has the advantages of nano-size and low immunogenicity. In a study, nanoparticles containing the secretome of MSC were synthesized and they demonstrated anti-osteoporotic effect via inhibiting the function of RANKL and promoting proliferation of osteoblasts and MSCs in ovariectomized rats. This novel treatment method showed a therapeutic effect similar to alendronate [117].

The Wnt pathway induces osteoblast differentiation and avoids adipogenesis, thus promoting bone formation. Sclerostin and DKK1 are secreted by osteocytes, binding to LRP5/6, blocking the Wnt pathway, and thus, enhancing bone resorption. Based on this fact, the monoclonal antibodies of sclerostin and DKK1 have been developed and shown improvement in BMD [118].

Recently, a study introduced the application of drug-loaded acrylamide hydrogels for potential use as vaginal rings, which contained acyclovir and demonstrated a cytotoxic effect to treat viral diseases. The newly designed hydrogels were featured by the nano-composition, which can increase the permeability, and increase cross-linker concentration, which can increase the absorption. This can provide a good model for the future design of novel design of drug delivery to treat osteoporosis [119].

Table 3 presents a comparison of the advantages and disadvantages in the treatments available for osteoporosis.

## 6. Discussion and Conclusions

Risk factors for osteoporosis include age, female sex, menopausal status, low calcium intake, and low physical activity. Estrogen promotes bone formation and suppresses bone resorption. Estrogen promotes the differentiation of osteoblasts through increasing serum level of OPG and TGFβ. The inhibition effect of estrogen on differentiation and activation of osteoclasts is via reducing RANK and M-CSF, and decreasing the response of osteoclast to RANKL. With the development of estrogen deficiency after menopause, disruption in the balance of bone remodeling process leads to progressive bone loss [15].

The most common cause of drug-induced osteoporosis is GIOP. The main cellular and molecular mechanisms involve altering in OPG/RANKL ratio to increase differentiation of osteoclasts, inhibiting the release of gonadotropin, reducing IGF-1, increasing DKK-1 to block the Wnt signaling pathway, and promoting the Notch signaling pathway to reduce the activity and differentiation of osteoblasts [68]. Compared with the past, early onset of menopause and ovarian dysfunction resulting from cancer treatments have become more and more common, which makes the risk of osteoporosis arise earlier, accompanied by more serious complications. Additionally, an increased risk of osteoporosis has also been observed in prostate cancer patients who undergo androgen deprivation therapy [64].

The immune system performs a critical role in regulating the signaling mechanisms of osteoporosis. Osteoporosis is a condition in which there is an imbalance between bone resorption and bone formation, leading to a decrease in bone density and an increased risk of fractures. This imbalance is influenced by a number of factors, including hormonal changes, genetic predisposition, and lifestyle factors.

The immune system is made up of a complex network of cells and signaling molecules that work together to protect the body from infection and disease. Some of the cells involved in the immune system, such as T cells and B cells, also perform a role in regulating the signaling pathways involved in bone remodeling. For example, T cells can produce cytokines, which are signaling molecules that can either promote or inhibit osteoclast and osteoblast activity. Some inflammatory cytokines, such as tumor necrosis factor-alpha (TNF-α) and interleukin-6 (IL-6), which are produced by immune cells during an inflammatory response, can directly or indirectly stimulate osteoclast activity and bone resorption, leading to bone loss. Chronic inflammation, such as that seen in rheumatoid arthritis or other autoimmune diseases, can therefore increase the risk of osteoporosis due to the prolonged presence of these inflammatory cytokines. On the other hand, some immune cells, such as regulatory T cells, can produce cytokines that have an anti-inflammatory effect and can inhibit osteoclast activity, thus promoting bone formation. The balance between pro- and anti-inflammatory cytokines is important for maintaining healthy bone remodeling.

In summary, the immune system performs a critical role in regulating the signaling mechanisms involved in bone remodeling and can contribute to the development of osteoporosis through its effects on cytokine production and inflammation. Therefore, strategies that aim to modulate the immune response, such as immune-modulatory therapies or lifestyle changes that promote immune system health, may be important in the prevention and treatment of osteoporosis.

Bone densitometry is often considered a tool to assist with the diagnosis of osteoporosis. However, some suggest that it may not be sensitive enough for early detection. Appropriate estrogen supplement and avoiding excessive glucocorticoid use are deemed the primary treatment for hormone-related and glucocorticoid-induced osteoporosis. Additionally, current medical treatment includes bisphosphonates, PTH, and RANKL inhibitors, such as denosumab. The therapeutic effect to retard bone loss is achieved by triggering apoptosis of osteoclasts, enhancing osteoblastogenesis and reducing differentiation of osteoclasts. Research investigating the effects of other dietary supplements, herbs, Chinese medicine, and lifestyle modification on osteoporosis are emerging in recent times [56,113]. However, potential risks of increasing cancer relapse must be considered when using estrogen replacement therapy in cancer patients; under such circumstances bisphosphonates may be regarded as the first line agents. Additionally, new treatment options with fewer side effects for combination therapy with estrogen have developed as well. On the other hand, autologous ovary transplantation after menopause with the ovarian tissue preserved in youth has been proposed. Although it seems technically and theoretically available, there is still lack of relevant data [15,65].

Inhibition of osteoclastogenesis is one strategy that has been explored for the treatment of osteoporosis. Osteoporosis is a disease that is characterized by the loss of bone mass and the deterioration of bone tissue, which can increase the risk of fractures. Osteoclasts are cells that break down bone tissue and perform a crucial role in bone remodeling. Inhibition of osteoclastogenesis can reduce bone resorption and, in theory, could lead to an increase in bone mass.

There are several drugs that target osteoclasts and their activity. For example, bisphosphonates and denosumab are two classes of drugs that are commonly used to treat osteoporosis. Bisphosphonates work by binding to bone and inhibiting osteoclast activity, while denosumab is a monoclonal antibody that targets a protein called RANKL, which is required for osteoclast differentiation and activity. While these drugs can be effective in reducing bone resorption and increasing bone density, they are not without their limitations. They may have side effects, such as gastrointestinal problems, musculoskeletal pain, and increased the risk of fractures in rare cases.

Furthermore, it is important to note that bone remodeling is a complex process that involves not only osteoclasts, but also osteoblasts, which are responsible for building new bone tissue. Inhibition of osteoclastogenesis alone may not be sufficient to improve bone health. Therefore, while inhibition of osteoclastogenesis is one strategy for treating osteoporosis, it may not be the best or only approach. A multifaceted approach that considers both bone resorption and bone formation may be more effective in improving bone health. This could include a combination of drugs that target both osteoclasts and osteoblasts, and lifestyle modifications, such as exercise and a healthy diet.

For individuals, there are several steps which can be used to prevent osteoporosis or detect it sooner:

1. Get adequate calcium and vitamin D: Adequate calcium and vitamin D intake is essential for maintaining bone health. Calcium-rich foods include dairy products, leafy green vegetables, and fortified foods, while vitamin D can be obtained mainly by sunlight, fatty fish, and egg yolks. The absorption of calcium is influenced by not only the concentration of calcium, but also the bioavailability of calcium. Several non-dairy foods contain factors, such as oxalic acid and phytic acid, that negatively affect the absorption of calcium by forming salts with low solubility, leading to a low absorption of calcium. Similarly, phytic acid in leafy green vegetables substantially reduces the absorption of calcium. In dairy products, calcium phosphates remain sparingly soluble salts and the absorption is further facilitated by casein [120]. Naturally calcium-rich waters are also considerable sources of dietary calcium due to the high bioavailability of calcium similar to dairy products [121].
2. Engage in weight-bearing exercises: Weight-bearing exercises, such as walking, running, and strength training, can help maintain bone density and reduce the risk of osteoporosis/fragility fracture risk.
3. Avoid smoking and excessive alcohol consumption: Both smoking and excessive alcohol consumption can contribute to bone loss and increase the risk of osteoporosis.
4. Consider bone density testing: Bone density testing, such as a dual-energy x-ray absorptiometry (DXA) scan, can help detect osteoporosis early and guide treatment decisions.
5. Discuss with a healthcare provider: Healthcare providers can provide guidance on lifestyle modifications, supplements, and medications that can help prevent or treat osteoporosis. In addition, individuals who are at an increased risk for osteoporosis, such as postmenopausal women and individuals with a family history of the disease, should take extra precautions to maintain bone health.

Overall, a proactive approach to bone health, including adequate nutrition, regular exercise, and regular monitoring of bone density, can help prevent osteoporosis or detect it sooner, allowing for earlier intervention and better treatment outcomes.

This current study has reviewed the related risk factors, cellular and molecular mechanisms, and therapeutic targets of primary and secondary osteoporosis. As the life expectancy lengthens, the proportion of elder population continues to grow, and the proportion of postmenopausal women will also scale up correspondingly. The population of both cancer survivors after cancer treatments and patients under long-term use of the aforementioned osteoporosis-related drugs expands accordingly. As the population who are vulnerable to osteoporosis continues to increase, the impact of osteoporosis on the overall human society will continue to extend. To understand the pathophysiology of osteoporosis, early prevention, diagnosis, and to start an appropriate and timely therapy is bound to remain as an important issue, which will greatly affect the health status of the entire human population and also determine the burden on society. However, many detailed cellular and molecular mechanisms underlying osteoporosis seem complicated and unexplored, and warrant further investigation.

## Figures and Tables

**Table 1:** The underlying mechanisms, pathways and related mediators of medications inducing osteoporosis.

Medications	Mechanisms to Induce Osteoporosis	Pathways/Related Mediators
**Glucocorticoids (GCs)**	promote the differentiation, proliferation, and activation of osteoclasts; suppress the differentiation of osteoblasts; induce the apoptosis of osteoblasts and osteocytes	RANKL, M-CSF, OPG, DKK-1, Notch pathway, TGFβ, IGF-1, SOD, CAT, caspase 3
**Aromatase Inhibitors** **(AIs)**	reduce the production of estrogen, leading to uncoupling of bone resorption and formation, increased differentiation of osteoclasts and decreased differentiation of osteoblasts	OPG, RANKL, M-CSF, TGFβ, IL-1, TNF
**GnRH agonists**		
**Chemotherapy**		
**Serotonin selective** **reuptake inhibitors** **(SSRIs)**		
**Medroxyprogesterone** **acetate (MPA)**		
**Proton pump inhibitors (PPIs)**	decrease intestinal calcium absorption	
**Antiepileptic drugs** **(AEDs)**	decrease intestinal calcium absorption; increase proliferation of osteoclasts	RANKL, DKK-1

**Table 2:** The timeline of different medications available for the treatment of osteoporosis.

**Bisphosphonates**	2000: Fracture risk reduction with alendronate in postmenopausal women 2000: Randomized trial of the effects of risedronate on vertebral fractures in postmenopausal women 2007: Effects of continuing or stopping alendronate after 5 years of treatment in postmenopausal women 2007: Once yearly zoledronic acid for treatment of osteoporosis in postmenopausal women
**Estrogen**	2002: Risks and Benefits of Estrogen Plus Progestin in Healthy Postmenopausal Women: Principal Results from the Women’s Health Initiative Randomized Controlled Trial
**RANKL inhibitor**	2009: Denosumab for prevention of fractures in postmenopausal women with osteoporosis 2017: 10 years of denosumab treatment in postmenopausal women with osteoporosis
**SERM**	2008: Efficacy of Bazedoxifene in Reducing New Vertebral Fracture Risk in Postmenopausal Women with Osteoporosis: Results from a 3-Year, Randomized, Placebo-, and Active-Controlled Clinical Trial
**PTH analogues**	2001: Effect of parathyroid hormone on fractures and bone mineral density in postmenopausal women with osteoporosis 2016: Effect of Abaloparatide vs. Placebo on new vertebral fracture in postmenopausal women with osteoporosis
**Sclerostine inhibitor**	2018: The foundation effect of Building bone with 1 year of Romosozumab leads to continued lower fracture risk after transition to Denosumab in postmenopausal women
**Cathepsin K inhibitor**	2014: The Effect of the Cathepsin K Inhibitor ONO-5334 on Trabecular and Cortical Bone in Postmenopausal Osteoporosis: the OCEAN Study 2016: Continuous Treatment with Odanacatib for up to 8 Years in Postmenopausal Women with Low Bone mineral Density: a Phase 2 Study

**Table 3:** A comparison of the advantages and disadvantages in the treatments available for specific conditions of high fragility fracture risk.

	Advantages	Disadvantages
**Bisphosphonates**	increase BMD in the femoral neck and lumbar spine in GIOP improve BMD in prostate cancer patients under ADT	risk of threatening fetal health nephrotoxicity risk of hypocalcemia risk of osteonecrosis of the jaw GI adverse events not totally eliminated when therapy is terminated
**PTH analogs**	increase BMD in lumbar spine and hip in postmenopausal women and men with GIOP lower osteoporotic fracture rates totally eliminated when therapy is terminated, lower risk in affecting fetal bone growth	risk of hypercalcemia should be avoid in patients with increased risk of osteosarcoma
**RANKL inhibitor**	lower osteoporotic fracture risk increased lumbar spine and hip BMD in GIOP	only subcutaneous delivery risk of hypocalcemia

## Data Availability

Not applicable.
